# Measuring trust in medical research: Perspectives from racial and ethnic communities underrepresented in research

**DOI:** 10.1017/cts.2025.40

**Published:** 2025-04-10

**Authors:** Sarah C. Stevens, Leo Valadez, Foujan Moghimi, Monica Guerrero Vazquez, Hailey N. Miller, Samuel Byiringiro, Cassia Lewis-Land, Roger S. Clark, Tosin Tomiwa, Joyline Chepkorir, Cheryl R. Himmelfarb

**Affiliations:** 1 Johns Hopkins Institute for Clinical and Translational Research, Baltimore, MD, USA; 2 Johns Hopkins University, Carey Business School, Baltimore, MD, USA; 3 Johns Hopkins University, Bloomberg School of Public Health, Baltimore, MD, USA; 4 Johns Hopkins University, School of Medicine, Baltimore, MD, USA; 5 Centro SOL, Baltimore, MD, USA; 6 Johns Hopkins University, School of Nursing, Baltimore, MD, USA; 7 Community Research Advisory Council of the Johns Hopkins Institute of Clinical and Translational Research, Baltimore, MD, USA

**Keywords:** Trust in medical research, community engagement, community-engaged research, underrepresented study populations, survey instrument evaluation, health disparities

## Abstract

**Introduction:**

Underrepresentation of diverse populations in medical research undermines generalizability, exacerbates health disparities, and erodes trust in research institutions. This study aimed to identify a suitable survey instrument to measure trust in medical research among Black and Latino communities in Baltimore, Maryland.

**Methods:**

Based on a literature review, a committee selected two validated instruments for community evaluation: Perceptions of Research Trustworthiness (PoRT) and Trust in Medical Researchers (TiMRs). Both were translated into Spanish through a standardized process. Thirty-four individuals participated in four focus groups (two in English, two in Spanish). Participants reviewed and provided feedback on the instruments’ relevance and clarity. Discussions were recorded, transcribed, and analyzed thematically.

**Results:**

Initial reactions to the instruments were mixed. While 68% found TiMR easier to complete, 74% preferred PoRT. Key discussion themes included the relevance of the instrument for measuring trust, clarity of the questions, and concerns about reinforcing negative perceptions of research. Participants felt that PoRT better aligned with the research goal of measuring community trust in research, though TiMR was seen as easier to understand. Despite PoRT’s lower reading level, some items were found to be more confusing than TiMR items.

**Conclusion:**

Community feedback highlighted the need to differentiate trust in medical research, researchers, and institutions. While PoRT and TiMR are acceptable instruments for measuring trust in medical research, refinement of both may be beneficial. Development and validation of instruments in multiple languages is needed to assess community trust in research and inform strategies to improve diverse participation in research.

## Introduction

The importance of representation of diverse populations in medical research is well-established [1–3]. Excluding or inadequately representing certain groups in medical research undermines the ultimate goals of such research to improve health and well-being. Underrepresentation in research can limit the generalizability of results; exacerbate health disparities and limit access to care; inhibit innovation due to homogeneity of treatment effects; hamper study enrollment and, subsequently, study success; and erode trust in the institution of medical research [4,5].

Evidence shows that despite awareness among researchers of the importance of diversity in study populations, disparities in participation persist. For example, in 2020, the FDA reported that of all clinical drug trials in the US, only 8% of the 32,000 participants were Black, 6% Hispanic, and 11% Asian [6]. Similarly, a 2020 review of Phase 1 oncology trials found 84.2% of the 3,197 participants identified as white [7]. These numbers do not reflect the US population, as approximately 42.1% of the US population identifies as a race or ethnicity other than non-Hispanic White [8].

The reasons for the continued underrepresentation of certain racial and ethnic groups in medical research are myriad, ranging from logistical challenges to a lack of awareness of and access to trials [5,9–11]. Of particular note is a general lack of trust in research, researchers, and the institutions that conduct research [12–14]. For example, a 2008 study of 717 individuals found that Black participants were more likely to distrust medical researchers than white participants on all trust domains tested [15]. This lack of trust likely stems from historical abuses of underrepresented research participants by the research community [5,11–13,16].

Since trust plays an important role in research participation, interventions to address distrust may improve the representation of historically marginalized groups in medical research [15,17–19]. Accurately measuring the success of such interventions to improve trust in medical research among underrepresented populations requires a suitable instrument. While multiple validated instruments for measuring trust in medical research exist, underrepresented racial and ethnic community perspectives on the instruments have not been reported [13,17,19–24].

Based on community-engaged research principles, our study aimed to identify a validated instrument suitable for assessing trust in medical research among Black and Latino communities to be used in future research studies. These communities were chosen given their historical and present underrepresentation in research and our team’s priority to improve trust and engagement in research among Black and Latino communities in and near Baltimore, Maryland. In Maryland, 12.4% of the population identifies as Black and 18.7% as Hispanic/Latino. As of 2022, immigrants comprise 16.7% of Maryland’s population and 70% of Latinos speak Spanish at home [25,26]. Thus, finding an instrument available in the Spanish language was also a priority.

## Methods

Study procedures were approved by the Johns Hopkins Medicine Institutional Review Board (IRB# 00383790) to ensure the project conformed to ethical guidelines. Participants signed informed consent documents before the start of discussions; informed consent methods are described below.

### Instrument selection

The Perceptions of Research Trustworthiness (PoRT) Scale and instrument from Measuring Trust in Medical Researchers (TiMRs) (Table 1) were selected for community review through the following process [23,24].

**Table 1.** Items in the perceptions of research trustworthiness (PoRT) and trust in medical research (TiMR) instruments

Perceptions of research trustworthiness (PoRT) scale [24]
Rate your agreement with the following statements: 1: Definitely agree, 2: Somewhat disagree, 3: Neither agree nor disagree, 4: Somewhat agree, 5: Definitely agree
Medical researchers tell people everything they need to know about being in a research study
If I had a chance to be in a medical research study, I wouldn’t be sure about being in medical research or not
Any info about me that I give to medical researchers would be kept confidential
Medical researchers would never give someone something that would hurt them, just to study how it works in people
Medical researchers keep dangerous things that could happen to people in a medical research study secret
Medical researchers try to hide any mistakes they make in their research studies
Participation in medical research benefits society
Medical research is secretly designed to give diseases to minority groups
Medical researchers usually tell people in a research study about different things they could do to get well
If I had a chance to be in a medical research study, it would be easy for me to decide to join in or not
Medical researchers only do research on people who know it is happening
Medical researchers would lie to people to convince them to be in a research study
Medical researchers are more interested in helping their own careers than helping people be healthy
My physician would not ask me to be in a medical research study if [they] thought it would hurt me
It is very likely that I, or people like me, will be used as guinea pigs in medical research
Medical researchers will share my personal info with anybody else they want to, even if I don’t tell them they can do that
I’m not sure that I have a voice in who can use my medical information
If I had a chance to be in a medical research study, I would be sure that participating in medical research would be the best choice for me
Measuring trust in medical research (TiMR) [23]
All things considered, how much do you trust medical researchers: none, a little, some, quite a bit, or a great deal?
When they are conducting research, how often do medical researchers have the best interests of participants from your racial or ethnic group in mind: never, rarely, sometimes, very often, or always?
How hard do medical researchers work to make sure that the participants in their studies are safe: not at all hard, a little hard, somewhat hard, very hard, or extremely hard?
How often do medical researchers tell participants everything they need to know about the risks of participating in their studies: never, rarely, sometimes, very often, or always?
How often do medical researchers treat participants from your racial or ethnic group the same as participants from other racial or ethnic groups: never, rarely, sometimes, very often, or always?
How hard do medical researchers work to make sure they keep information from participants private and secure: not at all hard, a little hard, somewhat hard, very hard, or extremely hard?
To what extent do medical researchers care more about their research than they do about the participants in their studies: not at all, a little, somewhat, quite a bit, a great deal?
When selecting participants for their most risky studies, how likely are medical researchers to select minorities: not at all likely, a little likely, somewhat likely, very likely, or extremely likely?
How often do medical researchers hide information about the possible risks of participating in medical research studies: never, rarely, sometimes, very often, or extremely often?
How often do medical researchers treat participants from your racial or ethnic group like guinea pigs in their studies: never, rarely, sometimes, very often, or extremely often?
How often do medical researchers want to know more than they need to know: never, rarely, sometimes, very often, or extremely often?

A literature review identified several validated instruments for measuring trust in medical research, defined as research that studies data from people to understand health and disease. Instruments broadly measuring trust in science were excluded, as were instruments that focused only on trust around healthcare provision (e.g., trust in patient–physician relationships). A selection committee, composed of academic and community stakeholders, reviewed the remaining eight instruments, all of which had demonstrated acceptable validity and reliability [13,17,19–24].

Five of those eight were excluded because they contained any items about healthcare provision and/or focused on physicians as medical researchers [13,17,20–22]. These decisions support our future aims to obtain feedback on trust in medical research generally and reflect the interdisciplinary makeup of the research teams at our institution. Of the remaining three, the instrument from Mainous et al. was omitted because it informed the development of PoRT and thus the key concepts from that tool would be represented by including PoRT instead [19,24].

### Instrument translation

While both PoRT and TiMR were validated with Black and Latino individuals, neither was validated in Spanish. Because our study included the Latino population, many of whom prefer speaking in Spanish [25], both instruments were translated to Spanish. The instruments were then back-translated into English and the forward and back translations were compared and revised to ensure fidelity of the final product [27].

### Study design, population, and recruitment

To gather community perspectives on the suitability of the two instruments, we organized focus group discussions with local community members. To be eligible to participate, individuals had to be 18 years of age or older, self-identify as Black or Latino, be fluent in English or Spanish, and be residents of Baltimore City or Baltimore County.

Participants were recruited by contacting individuals who had previously indicated interest in research opportunities with our institution. Potential participants whose preferred language was English received an interest email connecting to a REDCap survey that confirmed eligibility and collected preliminary scheduling information. A Spanish-speaking team member assisted Spanish-speaking participants in completing the interest form. Those meeting eligibility criteria were contacted by phone, provided a summary of the informed consent document, and invited to participate in the focus groups. Individuals that agreed to participate were emailed an informed consent form and demographics survey to complete via REDCap. Consent forms and demographic surveys were provided in the participants’ preferred language. Gift cards were provided as compensation for participation.

### Focus group procedures

Four focus groups were held between December 2023 and February 2024; two were conducted in English and two in Spanish. Each focus group had seven to ten participants. Based on preferences identified in the recruitment process, one focus group was held virtually in English via Zoom; the other three occurred in person.

Experienced qualitative researchers who were native speakers of the language of facilitation led the focus groups. Each focus group had one to two co-facilitator(s) and notetaker(s).

In each discussion, participants were given a copy of the first instrument to review, while the facilitator displayed it on the screen and read each item aloud. In one English and one Spanish discussion, PoRT was presented first. In the other two, TiMR was presented first. Before discussing the instrument, participants were asked to answer the following question, “If you had agreed to participate in research and you were handed this survey, do you feel that you could fill it out?” Response options were: Yes, with ease; yes, with some difficulty; I’m not sure; and no. Facilitators encouraged participants to share their perspectives on the instruments using the discussion guide that asked about the pros and cons of each instrument, with open-ended probing questions. Once the discussion about the first instrument concluded, the process was repeated for the second instrument. Finally, participants were asked to vote on which instrument they thought was better for measuring trust in medical research in Baltimore, MD. The response options were “PoRT,” “TiMR,” or “neither.”

### Data analysis

All focus groups were recorded and transcribed verbatim and translated from Spanish to English when needed. Transcripts were imported into Dedoose (Version 9.2) for coding and analysis. Initial codes were created based on the discussion guide (e.g., relevance to trust in medical research, clarity/comprehension of the wording). Additional codes were created based on the content of the discussion, after reading a sample of the transcripts. Two coders independently coded each of the four transcripts. All codes were subsequently reviewed by the two coders with discussion to reconcile any differences. The remaining disagreements were presented to the instrument selection committee for final consensus building. Thematic analysis was conducted on the final codes.

## Results

In total, 34 individuals participated in the four focus groups. The participants were largely female (79%) and highly educated, with 68% having at least some college education. About 38% of the participants identified as Black and about 56% identified as Latino. Table 2 describes additional demographic characteristics of the participants.

**Table 2.** Demographic characteristics of focus group participants

Category	Sub-category	No. of participants (%) *N* = 34
Sex	Male	7 (20.6)
	Female	27 (29.4)
Race	White	7 (20.6)
	Black/African American	13 (38.2)
	Asian	4 (11.8)
	Native Hawaiian or other Pacific Islander	2 (5.9)
	Self-identify	8 (23.5)
Ethnicity	Hispanic/Latino	19 (55.9)
Education level	Some high school no diploma	6 (17.7)
	High school diploma or GED	5 (14.7)
	Some college no degree	9 (26.5)
	Bachelor’s (4-year) degree	7 (20.6)
	Master’s	6 (17.7)
	Doctorate	1 (2.9)

Initial reactions to both surveys were mixed. Of the 34 participants, 15 (44%) said they could fill out PoRT with ease, compared to 23 (68%) for TiMR (Table 3). Overall, 74% said they preferred PoRT, while the remainder preferred TiMR (Table 3).

**Table 3.** Poll results from focus group discussions

	English 1 (N = 7)	English 2 (N = 8)	Spanish 1 (N = 10)	Spanish 2 (N = 9)	Total N (%)
First instrument presented in the group	TiMR^*^	PoRT^*^	PoRT	TiMR	
“If you had agreed to participate in research and you were handed this survey, do you feel that you could fill it out?”					
PoRT					
Yes, with ease	2	5	4	4	15 (44.1%)
Yes, with some difficulty	4	3	5	2	14 (41.2%)
I am not sure	1		0	1	2 (5.9%)
No	0		1	2	3 (8.8%)
TiMR					
Yes, with ease	4	4	9	6	23 (67.7%)
Yes, with some difficulty	3	4	1	0	8 (23.5%)
I am not sure	0		0	3	3 (8.8%)
No	0		0	0	0 (0.0%)
Which instrument do you recommend?					
PoRT	4	6	7	8	25 (73.5%)
TiMR	3	2	3	1	9 (26.5%)

* TiMR = Trust in Medical Research; PoRT = Perceptions of Research Trustworthiness.

**Figure 1.**
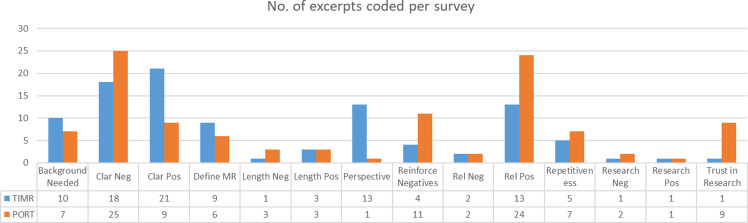
Number of excerpts per main codes by instrument. *TiMR = trust in medical research; poRT = perceptions of research trustworthiness.

These responses were supported by the discussion comments. The discussion generated four main themes: 1) relevance of the instrument to trust in medical research, 2) clarity or comprehension of the instrument, 3) potential for the instruments’ items to reinforce negative beliefs about research, and 4) comparison of the two instruments. Fig. 1 shows the number of excerpts coded by instrument, and Table 4 provides descriptions of the main codes.

**Table 4.** Codebook excerpt

Code	Description
Administration	Suggestions for successful administration of the instruments
Background needed	The instrument or questions only work for a specific audience or individuals with certain experience
Clar neg	There is confusion about the meaning of words, phrases, and/or the overall instrument
Clar pos	Words, phrases, and/or overall meaning of the instrument is clear and understandable
Define MR	Specifically refers to the clarity around the idea of “medical research”
Length neg	Discusses length of the instrument as a negative characteristic
Length pos	Discusses length of the instrument as a positive characteristic
Perspective	Discusses the “scope” of the questions/instrument. E.g. questions are about a group in general and not about the respondents’ personal perceptions/experiences
Reinforce negatives	The instrument or questions present medical research negatively
Rel neg	The instrument doesn’t measure trust in medical research or doesn’t do it well
Rel pos	The instrument measures trust in medical research
Repetitiveness	Either the questions or wording in the instrument are repetitive
Research neg	Participant describes their own negative experience with (medical) research
Research pos	Participant describes their own positive experience with (medical) research
Trust in research	Participant discusses trust in research generally (not a personal experience)

During these discussions, participants also shared substantially about their interactions with and beliefs about research, their negative healthcare experiences, and ideas about Black and Latino communities’ mistrust of medical research.

### Relevance of the instrument to trust in medical research

Overall, participants felt PoRT supported our research aims to assess trust in medical research.

“To make a point, I think this survey complies with the requirements for which we’re here. It informs you about the positive and the negative of it. Then we decide if we agree or not.” (Spanish)

“I get that they’re trying to determine whether they are suspicious about what medical researchers are doing and what they’re doing with your information and whether you can trust them.” (English)

Despite the overall positive response, there was some concern about PoRT’s ability to measure trust in medical research, which centered on missing elements.

“[PoRT] didn’t have much about understanding what the study is and why you’re part of it. I feel like [TiMR] had at least one question about understanding, while [PoRT] doesn’t really talk about that. I think that’s a helpful question to see if we understand what study we’re a part of.” (English)

TiMR also had several participants supporting its relevance, albeit fewer than PoRT did.

“[TiMR] is more concrete regarding the topics, yes. And I think that it contains language that we’re more used to when speaking, and that’s also important.” (Spanish)

“I think [TiMR] was geared toward getting the feel for what the Black community feels concerning medical research.” (English)

Several participants also shared concerns about the “perspective” or “scope” of the questions on TiMR.

“I felt like these questions don’t actually ask the individual their opinion about how much they trust research. It feels more about your ethnic group. I might not be aware of anything from my ethnic group, but I might be aware of something that happened to someone else’s ethnic group. That might still change my opinion and trust. Then I’m gonna answer this question about my ethnic group, and I don’t think it really gets at an individual’s level of trust.” (English)

“It’s like, “how often do medical researchers treat participants from your racial or ethnic group the same as participants from others?” I don’t know the answer to that. Like, “how often?” I have no idea. Do I feel like it’s different? Yeah. Maybe. I don’t know how often. It made it sort of an empirical “how many times is this happening” or sort of like a yes or no question, when it should be more of an opinion-based question.” (English)

### Clarity and comprehension of the instrument

The most frequent criticism of PoRT was confusion regarding question phrasing or meaning. While some comments were general (e.g., “I think it is kind of confusing. I’m not exactly sure what that means”), participants also named specific difficulties with items 2, 4, 17, and 18 on PoRT (see Table 1):

“I agree with everyone about all the stuff that they liked. But I think, for me, number two and four are a little convoluted. All the other ones are really easy to understand, but those ones I found myself rereading. Yeah, just a little bit complicated for language.” (English)

“I thought that 17 is a little weird. That it starts with, “I”m not sure.’ I think, I feel like if you’re not sure about something that’s sort of where number two and four as answer options come in. I think it would be better to just write the question as, “I have a voice and you can use my medical information,” and then people can decide if they are sure or not sure, kind of on the fence.” (English)

“I think number 18 is kind of confusing. I’m not exactly sure what that means.” (English)

On the other hand, some participants found PoRT easier to understand than TiMR because of PoRT’s standard agree-disagree Likert Scale response options.

“I think it’s easier to answer [PoRT]. You can run through [PoRT] quicker than [TiMR] because of the nuances of the answers to the previous questions [in TiMR]. This is a straightforward question. Your first answers are things that we’ve seen over and over again, so it makes it easier for you to complete it.” (English)

More comments were made in favor of TiMR’s clarity than PoRT. Participants described TiMR as “straightforward,” “more to the point,” and “an easy read.” However, feedback was mixed on the clarity of TiMR’s construct-specific response options.

“…You feel more comfortable responding because it’s very concrete. The answers are very concrete. As she said, you don’t have “possibly,” “maybe,” “it’s possible.’ It gives you more confidence to pick one of the answers.” (Spanish)

“[TiMR] was a little complicated because it mixed them up together. It seems as if every question had a different option to choose from.” (English)

#### Prior experience with research

Participants expressed a need for a specific background or prior experience to understand both instruments. First, they found the reading level to be a barrier to completing both instruments. Regarding PoRT, participants shared that “this was geared towards…educational level. This is the middle- to upper-level educational people who can understand these things” (English) and that “we need a little bit more knowledge to answer this question. People get confused in defining…the question that we have is ambiguous” (Spanish). For TiMR, the feeling was similar.

“But if the person can read a little bit—as <Name> was saying, there are people who finished school in their countries with great difficulty and didn’t have access to continue. It may be somewhat complicated for them.” (Spanish)

“It sounded like regular medical research-type questions. The tone felt like it was geared towards the <University> type people and not the regular street or neighborhood person to understand the nuances of the question. The tone is different.” (English)

Secondly, discussion on both instruments identified the need for some exposure to research to be able to answer the questions.

“Someone who isn’t familiar with the research phase would have a hard time filling out [PoRT].” (English)

“Who has the experience? Who would have an understanding of what a medical researcher would do with materials or how that medical researcher would select? I think you would have to have some experience with that environment in order to effectively be able to answer [TiMR]. You just wouldn’t know unless you have participated in a research study.” (English)

#### Defining medical research

Participants noted a lack of understanding surrounding the meaning of medical research and researchers. This sentiment was expressed in the discussions for both instruments across all focus groups.

“If you know nothing and not knowing what a medical researcher is, that stops you right there with being able to effectively answer any of those questions unless it’s something that you heard or someone shared with you or a rumor that has gone around in the community.” (English, PoRT)

“I myself am a medical researcher …when it asks specifically about medical researchers versus medical research, medical research as an institution, I feel differently about that than medical researchers. When they’re asking about medical researchers I am asked about my colleagues and myself. Perhaps how I feel that myself treats people of my ethnic group versus how my colleagues might treat people of my ethnic group, or other ethnic groups. That is different and that is directly in conflict…” (English, TiMR)

### Reinforcing negative perceptions of research

Participants in all focus groups mentioned that these instruments may reinforce or perpetuate negative ideas about research. This point was raised more frequently while discussing PoRT than TiMR.

“Personally, I think that [PoRT] creates more distrust regarding studies” (Spanish)

“I think it would get ’em suspicious. I think it will make you question the research. When I read that, the first thing I thought of is, why would they ask you these type of questions if it never happened? Did it ever happen, or did it not happen?” (English, PoRT)

“There are a lot of terms used here…terms such as lab rats, could manipulate the person and add feelings. I don’t think that this is the purpose of the survey…but if you’re using terms like lab rat, where does that leave us?” (Spanish, TiMR)

Despite the potential to reinforce negative perceptions, participants did believe measuring trust in medical research is important. While discussing TiMR, one participant shared, “Maybe they could have used different wording, but I definitely think I’m glad that they addressed the issue because we can’t pretend that that didn’t exist. It existed. To just overlook it or to pretend, that means it’ll continue to happen…we gotta address it and acknowledge it so we can move past it.” (English)

### Comparison of the PoRT and TiMR instruments

Across all groups, the same comparison theme was emphasized: PoRT is more relevant to the research goals, but TiMR is easier to understand. Several participants explicitly stated this comparison.

“I do like that the questions seem a little bit more clear [in TiMR], but I think they’re the wrong questions.” (English)

“I’m still with the same one. [PoRT] is to measure the trustworthiness that the Latino community has in medical studies. That would be the first one for me, if you want to measure trustworthiness. If we want to understand, then [TiMR].” (Spanish)

While all participants did vote for either PoRT or TiMR in the final poll, they continued to express that both instruments had aspects to improve upon. The following exchange summarizes the sentiment succinctly:

Moderator: “You chose one? Did you wanna elaborate on why you chose survey one [PoRT]?”

Participant: “There wasn’t a third choice.”

### Additional considerations

Although not the primary focus of discussion, participants had additional comments on the instruments regarding aspects such as the length and administration of the surveys.

#### Length

Participants generally found TiMR’s shorter length an asset. Further, some expressed not liking PoRT’s length.

“I think the one thing that I really like about [TiMR} is it doesn’t look overwhelming. With surveys, sometimes, they’re really long, or you don’t know that there’s multiple parts. This, you can just see what’s in front of you. You know that you’re done after 11 questions. I like that it’s pretty simple and not daunting.” (English)

“[TiMR] is shorter and to the point. I think that we didn’t like [PoRT] because it has more things and it’s more information, and maybe not everyone can receive as much information at the moment and analyze everything. I think that’s why [TiMR] is better. It’s shorter, and the topic is similar, and it’s more to the point.” (Spanish)

“They could reduce the number of questions and fix it a little bit visually. It looks very long, and people, when they see a lot, ignore it.” (Spanish, PoRT)

#### Repetitiveness

Participants criticized the repetitiveness of both instruments, although in different regards. For PoRT, they found the ideas duplicated across questions. “I think some of the questions are the same or similar but just other words” (English) and “for me, 18 and 10 I don’t know how to answer because they’re repeating the same thing” (Spanish). Regarding TiMR, they found it “redundant regarding discrimination and minorities” (Spanish).

#### Administration

Participants provided helpful suggestions about how the instrument(s) should be administered. They said it would be helpful to have an interviewer administer the survey so that they can explain the terminology. “If there is anything that you don’t understand, a word, a term, [the interviewer] can help him in that” (Spanish).

For TiMR, one participant also shared that the racial dynamics between interviewer and interviewee could affect responses. “Where it was talking about your ethnic group, I feel like if this was…a White person was giving me this I would be like, why is it my ethnic group? I was like, what exactly is “your”? Like who are you pertaining this to? Are you just giving this to me because I’m Black? If I was another, if I’m Asian, are you just giving this to Asians?” (English). They continued, “If me, as a Black person, if I was giving it to other Black people asking them how they feel about trust, but I feel weird giving this survey. This says your ethnic group, which means it’s not mine. If I was a Black person giving this to another Black person …To me, it should say “our” because I’m giving this to another Black person.”

Other participants suggested that the survey should be done using mixed methods, indicating that the instruments might raise emotional issues for some respondents and it would be helpful to give them a space to share those experiences. “…Maybe a space for the survey respondent to write if they—’cause if there are certain experiences that there’s something, maybe they have a chance to write that out. Maybe help contextualize their answers” (English).

## Discussion

The goal of this study was to select a preferred instrument to measure trust in medical research among Black and Latino residents of Baltimore, Maryland. Ultimately, in our community-engaged research initiatives that are implemented by multidisciplinary teams, not just physicians, we intend to use an instrument preferred by the community to measure trust. Community engagement in research is paramount in promoting trusting relationships[28] between researchers and the community [29,30]. Therefore, this study specifically aimed to collect community perspectives on existing instruments through focus groups and leverage that community input in the selection process.

Results were consistent across all four focus groups, regardless of discussion language. Participants found PoRT capable of measuring trust in medical research but expressed concerns about an “average” community member’s ability to understand the language. On the contrary, participants found TiMR to be more “straightforward,” but felt it wouldn’t capture an individual’s level of trust in medical research, which was our stated goal.

A major discussion theme among our instrument selection committee and focus group discussion participants was the intended measurement of trust. Many of the instruments in the literature are designed to measure trust between patient and healthcare provider or healthcare organizations, which is distinct from the field of medical research [31,32]. Even instruments that use more general language around medical research, referring to “medical researchers” instead of “your doctor,” for example, may not fully capture the intended area of trust due to undefined terminology. Specifically, the current literature does not differentiate between trust in medical research, medical researchers, and the organizations that facilitate medical research and, as noted by our participants, an individual may have differing levels of trust in these three entities. This observation may contrast with Hall et al, who found that “on the basis of exploratory factor analysis, pilot subjects did not differentiate trust in physician researchers from trust in medical researchers generally.”[17] However, that study did not establish if the concepts of the researcher are distinct from the institutions or field, and they go on to recommend that future validation efforts determine if distinctions exist in the trust measures of related constructs. Therefore, when choosing an instrument, it is important to clarify which entity is the focus of interest. It may also be beneficial to future research to develop an instrument that defines and measures trust in these three different entities.

Furthermore, the precise definition of “medical research” was a source of confusion among the participants, despite many having previously participated in medical research. Some participants specifically named the lack of clarity around the definition. Other participants demonstrated their confusion by sharing anecdotes they had with healthcare providers, even after being redirected by the facilitator back to the topic of research. It is, therefore, recommended that any administration of these or similar instruments contain an introduction clearly defining medical research. The definition should be easy to understand for those who have not participated in medical research, as determining levels of trust in those yet to, and potentially reticent to, participate in research is a critical step in implementing and evaluating initiatives to address disparities in research participation among Black and Latino communities.

In addition to confusion about the definitions of medical research, participants identified barriers to completing the instruments due to unfamiliar language and difficult-to-read phrases. Participants noted confusion both around the meaning of the questions (e.g., “I think number 18 [of PoRT] is kind of confusing; I’m not exactly sure what that means) and the words themselves (e.g., “Honestly, some of the words don’t coincide with my vocabulary”). Interestingly, despite participants expressing greater confusion with PoRT, it has a lower Flesch-Kincaid reading level than TiMR (10–12th grade vs College, respectively), although the reading level was higher than desired for both instruments. Since changing the wording would require re-assessing the instruments’ reliability and validity, strategies to promote respondent understanding should be engaged. Interviewer-assisted administration of these instruments may ameliorate some of the difficulties participants described with the language [33].

In the quantitative polls, our participants indicated a slight preference for PoRT over TiMR, despite the concerns about clarity and comprehension and mixed opinions on both instruments. These results are somewhat inconsistent with previous results from the validation of PoRT but consistent with the TiMR validation. (Note, the validation processes on both instruments included focus group discussions with Black and Latino individuals.) For PoRT, initial focus groups with Black, Latino, and White participants focused on content generation and defining dimensions of trust to develop the instruments, not on acceptability and clarity of the items.[24] Additional group discussions with 18 individuals assessed the clarity of each item of PoRT, and items were revised as a result of those cognitive interviews; however, the demographics of those individuals was not included in the publication [24]. TiMR validation also included cognitive interviews of participants from underrepresented racial and ethnic groups. However, Dykema reported some difficulty with comprehension. For example, “several participants said they did not have enough information to answer the trust questions, indicating they interpreted questions asking about their knowledge rather than for their evaluation.”[23] Dykema also reported some participants had challenges with the vocabulary of the instrument [23].

An additional consideration is that neither PoRT nor TiMR was validated in Spanish by the original authors, which is, incidentally, an example of the marginalization of some communities in medical research. While challenges with comprehension were reported in both our Spanish and English language discussions, some clarity issues with the spanish-spekaing population may have been identified and resolved if the instruments were validated in Spanish. A full validation of a Spanish-language instrument could yield an instrument more acceptable and comprehensible to Spanish-speaking populations.

Finally, our participants outlined the potential harm that administering these surveys could do by perpetuating negative ideas about medical research; this could be counterproductive to our ultimate aim of improving trust and participation in medical research among individuals from underrepresented racial and ethnic groups, particularly Black and Latino individuals. However, participants anecdotally expressed that the research was important and should be conducted. Administration of these instruments to participants should be coupled with education and engagement initiatives to promote awareness of and trust in research [34,35].

### Limitations

To reduce unnecessary burden and allow for ample discussion, each focus group was limited to reviewing two instruments. While PoRT and TIMR were selected through a thoughtful, criterion-based process to meet our future research aims, additional instruments are available that may be better-suited to the needs of other research teams. Reviewing additional instruments could have revealed alternatives preferred by our study population.

Due to our convenience sampling strategy, our sample was not representative of the population of Baltimore City and Baltimore County. Our participants had indicated willingness and interest in participating in research, with many of them having previously been in focus groups and studies. These participants may therefore be more trusting of research than the general Baltimore community, which could affect their perspective of the instruments. Those who had not previously participated in research may have had additional challenges in comprehension and/or be more critical of the instruments. Conversely, the experience with research may have provided more nuanced insights because they had more context to understand and compare the instruments.

The participants also had, on average, more education than the general population of Baltimore, which limits the generalizability of the results [36]. However, this may strengthen the argument that the language used in the instruments was too complex and would not be easy to understand for the average community member.

Additionally, the participants’ responses may have been biased by which instrument was presented first. We tried to limit the overall effect of this bias by presenting PoRT first in two of the groups and TiMR first in the other two. Although the sample was limited in size, we saw participants tend toward more critical comments on the second instrument presented. One participant even mentioned that she had a bad experience with research, so thinking through the first instrument had upset her and likely altered her frame of mind by the time she was reviewing the second.

## Conclusion

While PoRT and TiMR are acceptable instruments to measure trust in medical research in Black and Latino communities, there are opportunities to improve both scales. Specifically, simplifying the vocabulary may increase the accessibility of each instrument. Furthermore, PoRT could be more straightforward and TiMR could clarify the perspective, so respondents feel confident answering the questions. Finally, a definition of medical research provided at the beginning of the administration of these or similar instruments may assist participant’s comprehension. Those wishing to measure trust in medical research among underrepresented racial and ethnic communities may benefit from the development of a new instrument that is easier to comprehend.
